# Fibrodysplasia ossificans progressiva in Brazil: challenges and strategies to create assistance and educational networks

**DOI:** 10.1186/s13023-022-02503-6

**Published:** 2022-09-07

**Authors:** Alessandro Rozim Zorzi, Patricia R. Delai, Henrique L. C. Rosa, Wander E. Brito, Victor A. M. Montalli, Juliana C. Napimoga, Marcelo H. Napimoga, Francisco H. Nociti

**Affiliations:** 1 Center of Rare Diseases at Faculdade São Leopoldo Mandic (DoRa), R José Rocha Junqueira, 13, Campinas, SP 13045-755 Brazil; 2 Faculdade São Leopoldo Mandic Medical School, Undergraduate Medical Program, Campinas, SP Brazil; 3 Faculdade São Leopoldo Mandic, Instituto São Leopoldo Mandic, Laboratory of the Neuroimmune Interface in Pain Research, Campinas, SP Brazil; 4 Undiagnosed Diseases Network International (UDNI - Brazil), Piracicaba, SP Brazil

**Keywords:** Fibrodysplasia ossificans progressiva, Heterotopic ossification, Genetic diseases, Rare diseases

## Abstract

Fibrodysplasia ossificans progressiva (FOP) is an ultrarare condition and one of the most impactful disorders associated with progressive heterotopic ossification events. It is estimated that there are 120–150 patients in Brazil; however, currently, fewer than 100 patients have been identified, and the role of a FOP advocacy group (FOP Brazil) has been instrumental for the identification and follow-up of these individuals and families. The aim of this article is to summarize the current status of FOP in Brazil and describe strategies proposed to approach this challenge in a continental size country.

## The challenge

Fibrodysplasia ossificans progressiva (FOP) (OMIM 135100), an autosomal dominant genetic disorder of progressive heterotopic ossification (HO), is the most disabling disorder of extraskeletal osteogenesis in humans leading to the formation of an ectopic skeleton. FOP is caused by a recurrent heterozygous missense mutation in activin receptor IA (*ACVR1*), one of the seven type I TGFβ/BMP receptor family members, with most cases caused by a heterozygous gain-of-function mutation (c.617G>A; R206H) [1]. In individuals with FOP, HO initiates as a pathophysiological process recognized as flare-ups, which feature swelling, pain, erythema, and stiffness preceding overt bone formation [2]. One of the pioneer studies on the natural history of FOP was reported in 1993 by Cohen et al. [3], who reported that the average age for HO to occur was 5 years and that the most common sites of early HO were the neck, spine, and shoulder girdle. Approximately 80% of the responders had some restrictive heterotopic ossification by the age of 7 years, whereas at 15 years of age, approximately 95% presented with severely restricted mobility of the upper limbs. Later, Smith et al. [4] expanded our knowledge on the natural history of FOP, reporting that FOP patients had great toe deformities and that ossification in the large skeletal muscles began from birth to 16 years in most patients (25). Approximately 20 years later, Pignolo et al. [5] updated our knowledge on the natural history of flare-ups in FOP-affected individuals. Currently, it has been suggested that FOP may also be associated with a prolonged and hyperactivated immune response [6, 7]. Animal models have been instrumental not only in expanding our knowledge of the physiopathology of the disease but also in providing unique opportunities to advance treatment for FOP [8–10]. Some aspects make Brazil a unique country in South America, including its vast dimensions and population size, as well as its universal health care system. In Brazil, the challenge is to develop and propose strategies that will allow us to quickly identify the affected FOP population to avoid the numerous problems associated with its late diagnosis or lack of diagnosis [11]. In this context, as described below, we have addressed this challenge by proposing unique initiatives involving not only education, research and assistance but also by creating a task force composed of scientists and clinicians to support a bill that will make it mandatory for health care professionals to play a key role in the early diagnosis of FOP.

## FOP in Brazil

In 2016, the reported global prevalence of registered and confirmed FOP patients varied markedly from approximately 0.65 per million in North America, 0.47 per million in Western Europe, 0.27 per million in Latin America and 0.05 per million in Africa to nearly 0.04 per million in the Asia–Pacific region [12]. However, as the prevalence of FOP has been based on data from national FOP organizations, one should expect patients to exist outside the datasets, which in turn may explain the grand variability across the globe regarding the prevalence of FOP. In addition, one may speculate that medical care structures may also affect the ability of specific areas to efficiently diagnose FOP. Recently, Pignolo et al. [13] reported slightly higher estimates (0.88 per million) for FOP-affected individuals in the United States. In France, the current estimate of the prevalence based on the study of Baujat et al. [14] is higher, at 1.36 per million. The Brazilian population of FOP patients has been monitored by a FOP advocacy group, founded and managed by the patient’s family members, called FOP Brazil, which has spent significant resources to increase FOP awareness locally. In absolute numbers, Brazil currently has the third largest community of individuals with FOP, with 89 patients identified and registered in the FOP Brazil dataset (45 male and 50 female). However, a major drawback is that genetic diagnosis has not been available for the great majority of FOP patients in Brazil, where the diagnosis has been based on their clinical phenotyping: congenital malformations of the great toes and progressive HO in characteristic anatomic regions. Among the genotyped patients, most cases were the result of a de novo mutation, as there was no clinical or genetic evidence of FOP in their relatives. However, in at least one case, a family was identified with one parent and a child presenting the FOP phenotype, whose DNA sequencing analysis helped to identify ACVR1 activating mutation as the cause for FOP in 2006 [15].

**Fig. 1:**
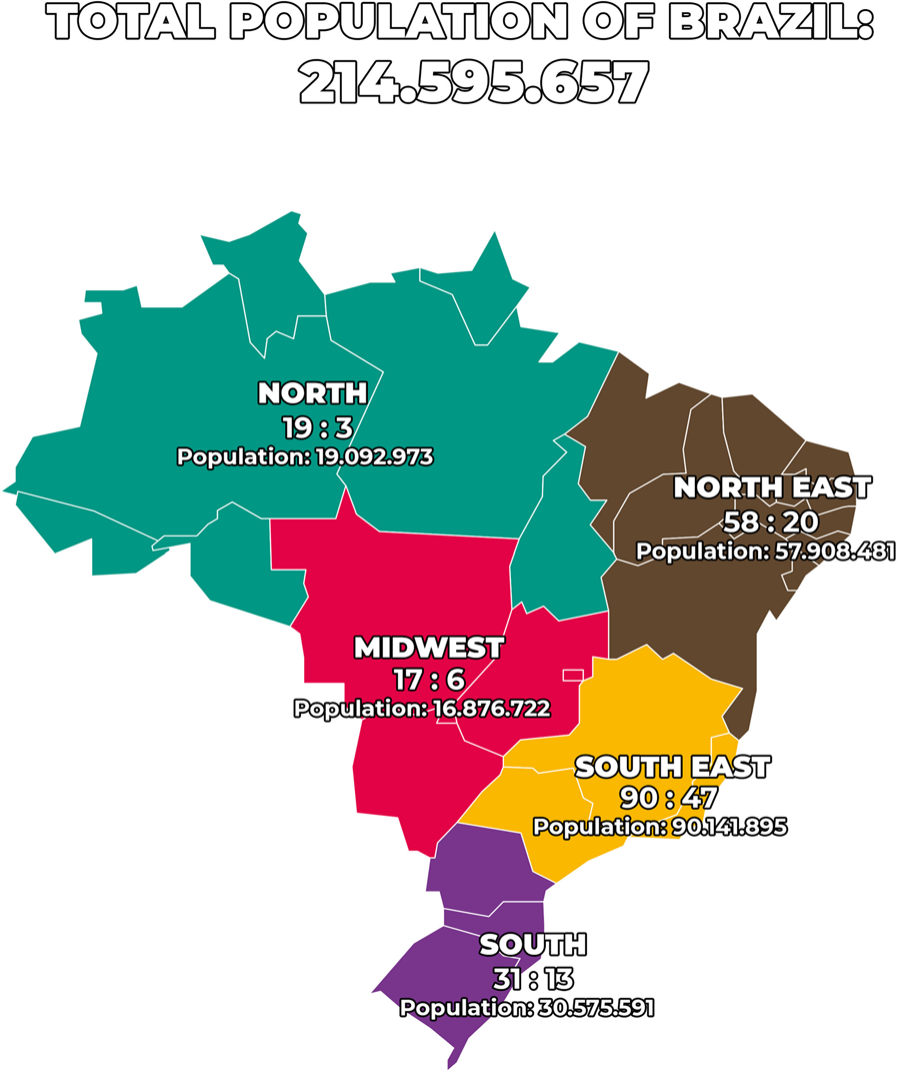
Demographic distribution of fibrodysplasia ossificans progressiva (FOP) in Brazil. The numbers on the map represent the number of expected cases based on the proportion of 1:1,000,000 [13] by the number of identified cases. Total population of Brazil and its distribution per state are based on the last national census (https://cidades.ibge.gov.br/brasil/panorama)

Based on the FOP Brazil dataset, it is possible to conclude that the largest population of identified FOP patients in Brazil is distributed across the southern states (≈ 67.4%), followed by the northeast states (≈ 22.5%), central states (≈ 6.7%) and the northern states (≈ 3.40). As the literature does not report a partiality of FOP in regard to any racial group or ethnicity, we speculate that it may simply reflect the fact that in such a large country, there are also very diverse and noninclusive health care settings. Although the Brazilian Universal Public Health Care System (SUS) was created in 1988 to guarantee equity in access to health care throughout the entire country, it is still recognized that the southern regions of Brazil, which feature the states of São Paulo and Rio de Janeiro as the major metropolitan areas, offer a more comprehensive health care system in general as well as stronger health care literacy. Figure 1 illustrates the demographics of FOP in Brazil according to the data provided by the FOP advocacy group in Brazil compared to the expected number of cases based on Pignolo et al. [13].

## Proposed strategies to improve assistance and literacy

Since the identification of the first case of FOP in Brazil in 2000, there have been isolated and individual initiatives to create reference centers to foster assistance and literacy, including the foundation of the advocacy group FOP Brazil in 2004 (http://www.fopbrasil.org.br). If we take as true the apparent prevalence rate of approximately one per million individuals reported by Pignolo et al. [13], in Brazil, we are missing a significant number of patients with FOP, and therefore, there is an urgent need to improve our strategic approaches to diagnosing FOP. To overcome our local challenges, we have proposed actions at three main levels: (1) promote early diagnosis, (2) disseminate information on FOP to health care professionals, and (3) create collaborative research initiatives with consolidated groups across the globe to approach gaps in knowledge regarding the impact of FOP on affected individuals and their families. With regard to being able to diagnose FOP as early as possible, we have partnered with the Federal Government and Brazilian Congress to pass a bill that will make it mandatory for health care professionals involved with the early care of newborns to screen them for great toe deformities [4] as part of the screening tests for other genetic conditions that are already in place [16]. Once the presence of great toe deformities is identified by this initial screening, these families will be directed to FOP reference centers for genotyping and further clinical examination. To our knowledge, this is the first time anywhere in the world that newborn screening specifically focusing on the potential early identification of FOP has been proposed before they even have any symptomology associated with the disease, which is typically diagnosed a few years after the first symptoms. The approval process for this new approach is about to be completed in the Brazilian Congress with the support of many people, and we anticipate it will be part of our routine at some point in 2022, with true potential to impact FOP-affected individuals’ quality of life, as well as that of their family members. The second initiative that we have already put in place refers to increasing literacy among health care professionals. At the Medical and Dental School—São Leopoldo Mandic (Brazil), we created a core with selected faculties involved in the study of rare and ultrarare conditions to propose initiatives related to disseminating information on FOP among health care professionals. As the first step, and perhaps the most challenging one, FOP is now officially listed as a separate and specific subject in different disciplines across the Medical and Dental School curricula. Students will have the opportunity to explore content specifically relevant to FOP not only in the context of theory but will also interact with FOP patients. At its initial stages, we already noted an increasing interest in a deeper understanding of the disease as well as a gradual search for research-assisted initiatives by medical and dental students. Such an initiative is also extended to the postgraduate level, where current physicians are gathering in-depth information about FOP. Last, with a real and immediate need for expanding our knowledge on the implications of FOP for affected individuals, we have established a global research network with well-known research groups worldwide, including the groups at the University of Pennsylvania, Western University and University of California at San Francisco.

Currently, there is a lack of information regarding the prevalence of FOP and how to assist people with this condition in other South American countries, so we hope our initiative will serve as a trigger to create educational and research networks in South America to allow us to design specific collaborative health care programs that will better assist this population and connect the scientific community in the Southern Hemisphere. Therefore, the goal of this group is to identify resources to support FOP-related research and to understand the functions of ACVR1 in normal and abnormal craniofacial/dental development and function and to use that knowledge to guide interventions, improve diagnostic strategies, and increase the quality of life of individuals affected by FOP. Each arm of this “tripod” strategy is flexible and will continue to adapt its goals as emerging evidence becomes available and as new adjustments are needed depending on regular survey outcomes. We intend to evaluate the impact of these initiatives based on our ability to (1) identify new FOP patients and improve our communication with the patient’s family, (2) generate new and relevant scientific information and (3) recruit and retain health care professionals interested in assisting FOP-affected individuals as well as their close relatives.

## Conclusion

A new era has begun in Brazil for patients with FOP with the development of policies to allow early diagnosis as well as to improve literacy and disseminate knowledge among health care professionals. We anticipate that these initiatives will serve as catalysts for research funding, education and assistance.

## Data Availability

Not applicable.
